# Solitary Necrotic Nodule of the Liver: A Report of Two Cases and Review of the Literature

**DOI:** 10.1155/2011/845406

**Published:** 2011-07-31

**Authors:** Hong-Qun Wang, Zheng-Sheng Wu, Dao-Wang Li

**Affiliations:** ^1^Department of Pathology, The Third People's Hospital, Hefei 230022, China; ^2^Department of Pathology, Anhui Medical University, 81 Meishan Road, Hefei 230032, Anhui Province, China; ^3^Department of Urology, The 105th Hospital of People's Liberation Army, Hefei 230011, China

## Abstract

To investigate the clinicopathological characteristics and possible causes of solitary necrotic nodule of the liver (SNN), two cases of SNN of the liver were studied with clinicopathological data, immunohistochemistry, and histochemistry staining. The patients had no specific symptoms, with negative results for the serum tumor markers. CT and ultrasound all showed low-density lesion. Morphologically, there was isolate, single necrosis tubercle of the liver. It was composed of a central necrotic core and a peripheral fibrotic capsule with inflammatory cells, including histiocytes, plasma cells, lymphocytes, and so forth. The staining result of PAS, acid-fast, and iron was all negative, and AG + VG staining showed that the outline of reticular fibers and collagen was intact. Vimtin was positive for necrotic tissue and surrounding fibrous tissue. CD34 and CD68 was both positive for case 1. CK was negative in case 2 but positive for a few residual cells in case 1. SNN of the liver is a rare nonmalignant disease with a good prognosis. Hemangioma and fatty liver might be ones of the causes of SNN.

## 1. Introduction

Solitary necrotic nodule (SNN) of liver is a rare benign disease which was first described by the Shepherd and Lee in 1983 [1] and few SNN cases has been reported in the literatures. During 2006–2009, we encountered 2 cases SNN in the Department of Pathology of the First Affiliated Hospital of Anhui Medical University and of the Third People's Hospital of Hefei. With literatures review, we investigated the clinical and pathological features and possible etiopathogenesis.

## 2. Case Reports

**Figure 1:**
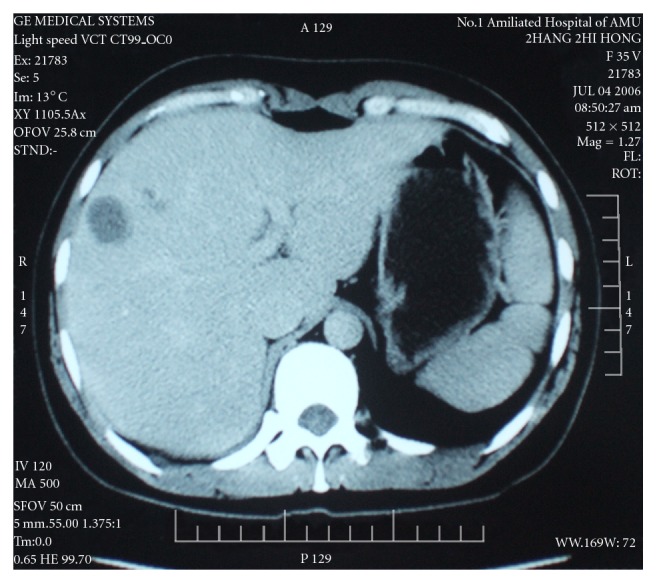
CT showed one low-density lesion under the right hepatic lobe capsule for case 1.

**Figure 2:**
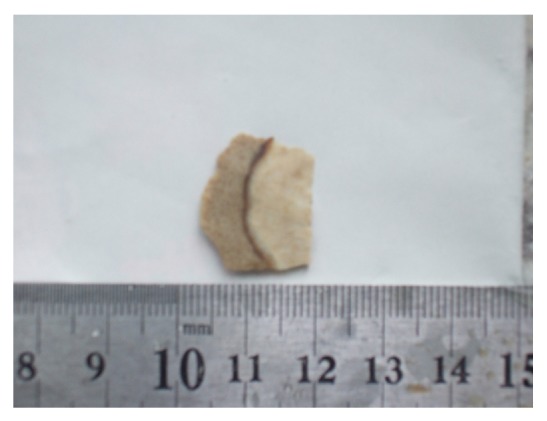
There was a nodule with clear boundary into the gross specimen.

Case 1, female, 35 years old, no obvious symptoms, SNN was accidentally found in 2006 through physical examination, all the parameters by laboratory tests were all normal, including routine blood test, serum electrolytes, liver function, and serum twelve tumor markers. HAV, HBV, and HCV were all negative. Computer Tomography (CT) showed multiple small hemangiomas of the patient's right hepatic lobe and one low-density lesion under the right hepatic lobe capsule (Figure 1). B-ultrasound showed the hypoechoic lesion with inhomogeneous internal echo. The lesion and around 2-3 cm liver was surgical resection. Gross examination: there was a 4 cm × 3 cm × 3 cm sized gray nodule into the part liver with the size of 5 cm × 5 cm × 3 cm, which boundary was clear with around congestive hemorrhage zone (Figure 2). The other send two pieces of tissue with purple-brown cut surface which size was 1.2 cm × 0.8 cm and 0.5 cm × 0.5 cm, respectively, were both diagnosed hemangiomas.

**Figure 3:**
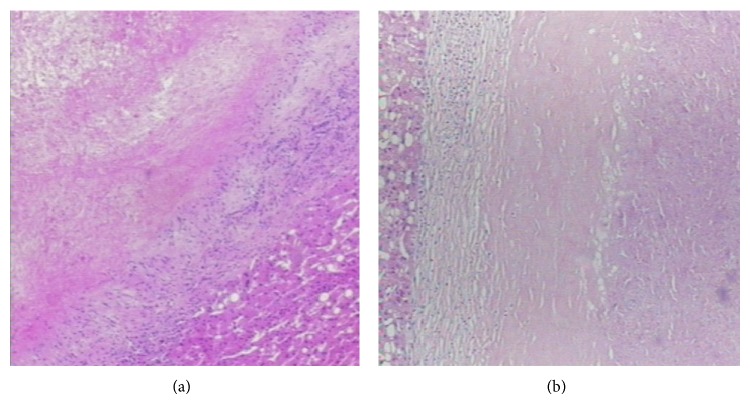
The two patients both had the typical image of SNN (HE × 40). (a) Case 1; (b) Case 2.

Light microscope: the central part of nodule was eosinophilic coagulation necrosis lesion which could be seen a small amount of cell ghosts of remaining liver cells and multinucleated giant cells. In the meanwhile, the stove gathered inflammatory cells could also be seen, which included lymph cells, histocytes, neutrophils, and plasma cells. Fibrous tissue wrapped around the necrotic area, in which fibrous tissue produced hyperplasia with hyaline degeneration, accompanying infiltration of inflammatory cells including small-to-moderate amount of lymphocytes, and some eosinophils, plasma cells, monocytes, and multinucleated giant cells (Figure 3(a)). The liver tissue surrounding the nodule were mild edema, cholestasis, and steatosis. Liver sinus filled the blood. Fibrous tissue of portal area was mild hyperplasia with a few of lymphocyte infiltration. Sublobular and interlobular veins were expansion.

**Figure 4:**
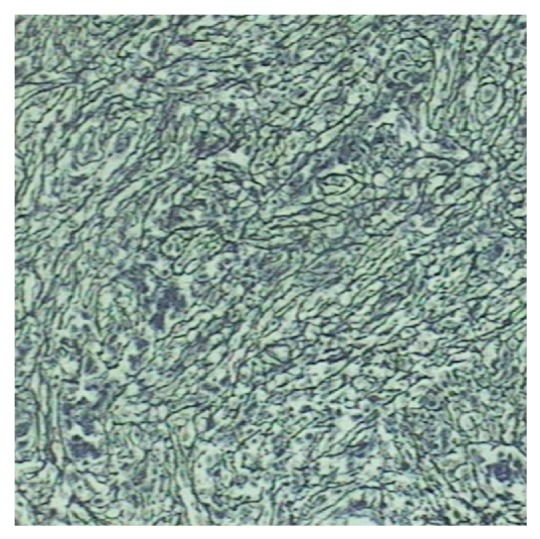
Ag + VG staining showed that the outline of reticular fibers and collagen was intact (×100).

**Figure 5:**
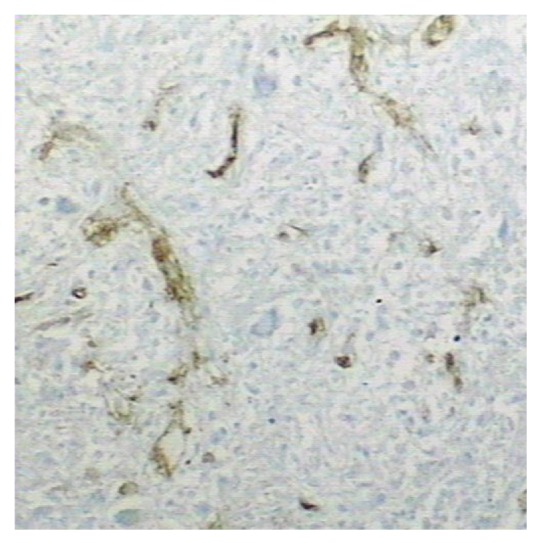
CD34 was positive for case 1 (MaxVision ×100).

Immunohistochemistry and histochemistry staining: periodic acid-Schiff reaction (PAS) was negative, negative for acid-fast staining and iron staining, and Ag + Van gieson (Ag + VG) staining showed that the outline of reticular fibers and collagen was intact (Figure 4). Vimtin (Vim) was positive for necrotic tissue and surrounding fibrous tissue. CD34 was positive for a great number of blood vessels ghosts (Figure 5), CD68 was diffuse positive. Cytokeratin (CK) was positive for a few residual cells. Because CT showed low-density lesion for the case 1 and the lesion was suspected malignancy. Intraoperative frozen section was done which showed coagulation necrosis. HE section was diagnosed for SNN. The patient recovered well after surgery with no recurrence during 36-month followup. 

**Figure 6:**
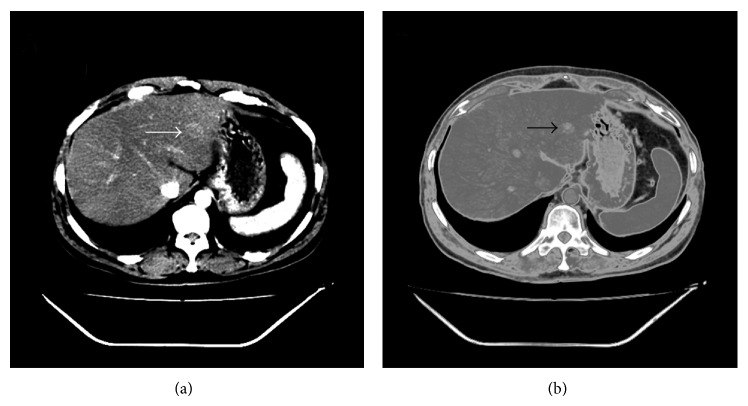
CT showed one lesion in the left lobe liver of case 2 (arrows). (a) contrast enhancement imaging; (b) delayed imaging.

Case 2, male, 46 years old, due to traumatic right rib fractures, one lesion was found in the left lobe of the patient's liver through CT and B-ultrasonography in 2008 (see Figure 6). Laboratory examinations including routine blood test, electrolytes, liver function, and twelve tumor markers were normal. HAV, HBV, and HCV were negative, and HbsAb was weakly positive. The patient's serum lipid was high: triglyceride (TG) 4.24 mmol/L (normal value 0.4–2.00 mmol/L), total cholesterol 7.48 mmol/L (normal value 3.00–5.70 mmol/L), low-density lipoprotein and very low-density lipoprotein were higher than normal ones (4.59 and 1.93 mmol/L, resp.). The patient with type 2 diabetes and alcohol abuse had been for many years. CT scan showed liver parenchyma density diffusely reduced. Color Doppler ultrasound showed that the liver left lateral lobe was seen somewhat lower echo mass with the size of 1.6 cm × 1.5 cm, clear boundary, posterior echo enhancement accompanying heterogeneous internal echo. In the meanwhile, the patient was showed fatty liver. 

During the operation, the left lobe liver looked slightly hypertrophic, accompanying surface concave, a mass with approximately 2 cm in diameter was seen. The mass and around 2-3 cm liver was resected. Gross examination: there was one grey red irregular liver tissue with the size of 4.5 cm × 3.0 cm × 2.5 cm. A 1.8 cm gray nodule on the section with clear boundaries and qualitative toughness was observed. Light microscope: the central nodular area was eosinophilic coagulation necrosis, in which there were fat ghosts and cholesterol crystals. The surrounding fibrous tissue was hyperplasia with hyaline degeneration, in which moderate amount of lymphocytes and a small amount of plasma cells, eosinophils granulocyte, infiltrated. There was narrow homogeneous hyaline zone between fibrous tissue and necrotic areas (Figure 3(b)).

The liver tissue surrounding the nodule was mild edema, cholestasis, and notable steatosis. Fibrous tissue of portal area was mild hyperplasia with a few of lymphocyte infiltration. Sublobular and interlobular veins were expansive. Immunohistochemistry and histochemistry staining: the staining result of PAS, acid-fast, and iron was all negative, AG + VG staining showed that the outline of reticular fibers and collagen was intact (Figure 4). Vim was positive for necrotic tissue and surrounding fibrous tissue. CD34 was focal positive, CD68 was positive for a few cells. CK was negative. Because CT showed one lesion of the liver for case 2 that was suspected malignancy, intraoperative frozen section was done which was described as coagulation necrosis; HE section was also diagnosed for SNN. The patient recovered well after surgery, and no recurrence was done during 34-month followup.

## 3. Discussion

Solitary necrotic nodule of liver is an uncommon disease and often would be easy to being diagnosed as a malignant metastatic disease. Most patients had no obvious symptoms, frequently were found out by other diseases, and stumbled to a hospital for examination or physical examination findings. SNN reported in the literature mostly was under the capsule, a small number in the liver parenchyma, and most of patients were located in the right lobe and the minority in the left lobe. The majority of patients were single nodule, and the minority were nodules. Average diameter was about 2 ~ 3 cm; Serum CA-19, CEA and other tumor marker test results were normal [2–4]. In our cohorts of patients, one was in the left lobe of the liver parenchyma, the other one was in the right lobe under the liver capsule, and the nodule diameter was 1.8 cm and 4.0 cm, respectively. The 2 cases' serous tumor markers were normal which consisted with the literature. And the 2 cases' liver function was normal with no hepatitis virus infection.

Microscopically, all patients all had the typical image: coagulation necrosis of the center part and the surrounding fibrous membrane composed SNN; the fiber membrane accompanied inflammatory cells infiltration including histocyte, lymphocytes, plasma cells, eosinophils, and so forth. PAS, Grocott and acid-fast staining all showed no bacterial, fungal, and parasitic infections [1–4]. In current study, the cases both had the typical image of SNN, PAS, and acid-fast staining was also negative.

The cause of SNN is uncertain and maybe diverse: SNN might be potentially representing the evolutionary process from various benign diseases, such as infectious abscess, parasitic granuloma, hemangioma (or blood-borne disease), benign tumor, hematoma, trauma, and other benign disease development; about 50% of cases could be found with other types of tumors [1–6]. Immunohistochemistry and histochemistry staining of our 2 cases showed intact reticular fibers. Vim and CD68 were both positive, accompanying fibrous tissue generation, which all suggested that macrophages phagocytosed the necrotic tissue during the necrotic process and accompanied fibrous tissue generation. The liver tissue surrounding the nodules tissue were mild edema, varying degrees of cholestasis, and steatosis, fibrous tissue of portal area were mild hyperplasia with lymphocytes infiltration, sublobular and interlobular veins expanded, which all suggested the lesion and the surrounding liver tissue was hypoxia and ischemia with varying degrees of inflammatory response. 

In this cohort of cases, the case 1 accompanied multiple hepatic hemangiomas; the sinusoidal around the lesions of SNN was expanded and full of blood. On the necrotic nodules, there were a lot of small blood vessels ghosts (CD34 positive), focal inflammatory cells aggregated, and CD68 was diffusively positive. We speculated that the formation of the patient's SNN closely associated vascular tumor, alike the Berry [5] reporting. We supposed that hemangioma hardening, degeneration and necrosis leaded reacting inflammatory cell infiltration, phagocytosis, and wrapping necrotic tissue, in the meanwhile, the surrounding fibrous tissue was hyperplasia, so the characteristic necrotic nodules was formatted.

The case 2 showed fatty liver through ultrasound examination. The patient's blood lipid significantly increased. There was obvious fatty degeneration of liver cells around the lesion. In the necrotic nodule, fatty vacuoles blur and cholesterol crystals had been observed, while lobular and interlobular veins around the SNN significantly expanded, which were possibly related to fatty liver, hypoxia, and ischemia, but that has not been reported in the related literature. So, we still need to further observe and study in larger sample cases. At the same time, the patient had no vascular tumor, and being different from case 1, which suggested that SNN maybe have several causes.

In the CT imaging, both the cases showed that low-density lesions and ultrasound suggested low hypoechoic changes. Literatures reported that most patients' ultrasound showed hypoechoic lesions, or accompanying a slight edge of high echo, and all patients were nonhomogeneous echo [2, 4, 7]; CT scan of SNN was the low-density lesions, in which a few of cases delayed imaging showed slight edge enhancement [7].

SNN is a rare disease which has not obvious symptoms and potential complications, in the meanwhile, it is not obvious influence on the health [2]; clinicians should give right diagnosis through clinical features combining imaging examination and experimental inspection results. Try best to avoid surgical treatment which reduces unnecessary trauma; the best treatment is conservative treatment and followup.
